# Research on IMU Calibration Model Based on Polar Decomposition

**DOI:** 10.3390/mi14030697

**Published:** 2023-03-21

**Authors:** Guiling Zhao, Maolin Tan, Xu Wang, Weidong Liang, Shuai Gao, Zhijian Chen

**Affiliations:** School of Geomatics, Liaoning Technical University, Fuxin 123000, China

**Keywords:** IMU, calibration, polar decomposition, nonorthogonal error, misalignment error, installation error model

## Abstract

As an important deterministic error of the inertial measurement unit (IMU), the installation error has a serious impact on the navigation accuracy of the strapdown inertial navigation system (SINS). The impact becomes more severe in a highly dynamic application environment. This paper proposes a new IMU calibration model based on polar decomposition. Using the new model, the installation error is decomposed into a nonorthogonal error and a misalignment error. The compensation of the IMU calibration model is decomposed into two steps. First, the nonorthogonal error is compensated, and then the misalignment error is compensated. Based on the proposed IMU calibration model, we used a three-axis turntable to calibrate three sets of strapdown inertial navigation systems (SINS). The experimental results show that the misalignment errors are larger than the nonorthogonal errors. Based on the experimental results, this paper proposes a new method to simplify the installation error. This simplified method defines the installation error matrix as an antisymmetric matrix composed of three misalignment errors. The navigation errors caused by the proposed simplified calibration model are compared with the navigation errors caused by the traditional simplified calibration model. The 48-h navigation experiment results show that the proposed simplified calibration model is superior to the traditional simplified calibration model in attitude accuracy, velocity accuracy, and position accuracy.

## 1. Introduction

The inertial measurement unit (IMU) is the core sensor of the strapdown inertial navigation system (SINS). Its measurement accuracy is directly related to the navigation accuracy of SINS. The IMU manufacturing and installation errors cause navigation errors to accumulate over time [1,2]. The IMU calibration model is a mathematical relationship that reflects the sensor errors and environmental factors. Establishing a suitable IMU calibration model is a key technology for error compensation [3,4]. Therefore, it is necessary to establish an IMU error compensation model which meets the accuracy requirements and calibrate it accurately to improve the SINS accuracy [5,6,7]. The IMU mathematical model is divided into the static mathematical model, dynamic mathematical model, and random mathematical model. This paper mainly studies the static mathematical model.

The machining and assembly processes cause the sensitive axis of the gyroscope and accelerometer not to coincide with the carrier coordinate system axis [8,9]. This leads to the installation error. In the navigation-grade SINS, the installation error is a very important parameter that affects the navigation output accuracy. In [10], the SINS carries out the static navigation experiment. The results show that the attitude and velocity errors are significantly reduced after compensating for the installation error. In [11], the influence of a fiber-optic gyroscope (FOG) installation error on the attitude heading reference system (AHRS) is simulated and analyzed. The results show that the attitude error caused by the gyroscope installation error is related to the carrier motion environment. The more intense the carrier motion, the bigger the attitude error. What is more serious is that if the carrier is in a rocking state, the installation error of the gyroscope will also stimulate other errors in the system. For the installation error of the accelerometer, the more severe the linear motion, the more obvious the velocity error caused by the accelerometer installation error.

Some researchers find a coupling between the accelerometer installation error and the gyroscope installation error. In [12], it derives an IMU calibration model based on the velocity error. Based on the observability analysis method, it is found that there are three coupling relationships in the installation error parameters. The pulse output of the accelerometer is added to solve the problem that the installation error parameters cannot be fully identified. In fact, if the velocity and the attitude errors are used as observations at the same time, this coupling relationship will disappear. In [13], it proposes a method of constraining the carrier coordinate system. Assume that the *x*-axis of the carrier coordinate system coincides with the *x*-axis of the acceleration coordinate system. This assumption can reduce the number of installation error angles. At the same time, the coupling between the accelerometer installation error and the gyro installation error is eliminated. However, ref. [13] only studies the calibration scheme. It does not evaluate the impact of the simplified model on the navigation accuracy of SINS.

Some researchers calibrate the navigation-grade SINS by simplifying the installation error angles. In [14], it proposes a hybrid calibration scheme aiming at the high-precision FOG IMU and ring laser gyroscope (RLG) IMU. It reduces the installation error angles of the gyroscope calibration model from six to three. In [15], the ultrahigh precision IMU calibration model is studied. Additional g sensitivity errors, accelerometer cross-coupling, and lever arm errors are introduced. The model includes g sensitivity error, accelerometer cross-coupling error, and lever arm error. Although the gyroscope installation error is simplified, the filtering dimension is also as high as 51. In [16], the gyroscopes and the accelerometers are almost orthogonal. It is defined as the *x*-axis of the inertial sensor assembly (ISA) coinciding with the *x*-axis of the platform coordinate system. In this way, the transformation matrices of the platform coordinate system relative to the accelerometer coordinate system and the platform coordinate system relative to the gyroscope coordinate system are simplified to lower triangular matrices. In [17,18], one axis of the accelerometer coordinate system is assumed to coincide with one axis of the carrier coordinate system. Thus, the installation error of the accelerometer assembly is represented by three small angles, and the installation error of the accelerometer assembly is expressed with three small angles. The accelerometer installation error matrix is simplified as a lower triangular matrix to achieve a certain constraint. In [19], the multiposition calibration method is optimized for the IMU nonlinear scale factor. However, in the deterministic error model, the installation error number of the gyroscope and accelerometer are all simplified to three. In [20,21,22,23,24], the installation error of ISA is also simplified. In commercial grade or tactical grade SINS, simplifying the IMU installation error can meet the requirement of system accuracy. Except for high-precision navigation-grade SINS, the simplified installation error model has a serious impact on system accuracy. So, the indepth analysis of the installation error matrix is important in engineering applications. This paper mainly focuses on the following problems. How to describe the installation error of inertial sensor assembly by mathematical model? What are the geometric characteristics of the installation error matrix? How does the simplified installation error matrix affect the navigation system?

This paper introduces the polar decomposition [25] to decompose the IMU installation error matrix. Through a series of matrix decompositions and equivalent transformations, the mathematical calibration model is established. The installation error is decomposed into a nonorthogonal error and a misalignment error. The installation error matrix is finally decomposed into a symmetric matrix and an oblique symmetric matrix. In order to further analyze the results after decomposition, three sets of IMU are calibrated by a three-axis inertial-test turntable. We analyze the nonorthogonal and misalignment errors by introducing infinite norm and two-norm. The analysis results show that the misalignment error is larger than the nonorthogonal error. This result is helpful to improve the production and assembly of IMU. For the SINS in which the misalignment error is larger than the nonorthogonal error, a new simplified model of installation error is proposed. This simplified model is verified by 48 h navigation experiments. The navigation accuracy of the proposed model is better than the traditional simplified model in attitude, velocity, and position.

The paper is organized as follows. Section 2 is an installation error analysis and modeling. In Section 3, polar decomposition is introduced to decompose the installation error matrix. The installation error model, based on polar decomposition, is obtained. This method is also shown in geometric space. In Section 4, three sets of IMU are calibrated by a three-axis inertial test turntable. The characteristics of the nonorthogonal error and the misalignment error are analyzed. Section 5 presents a new simplified model of the installation error. The proposed simplified model and the traditional simplified model are compared in navigation simulation experiments. Section 6 is the conclusion.

## 2. Installation Error Model of the IMU

The frames used in this paper are provided in Table 1.

**Figure 1:**
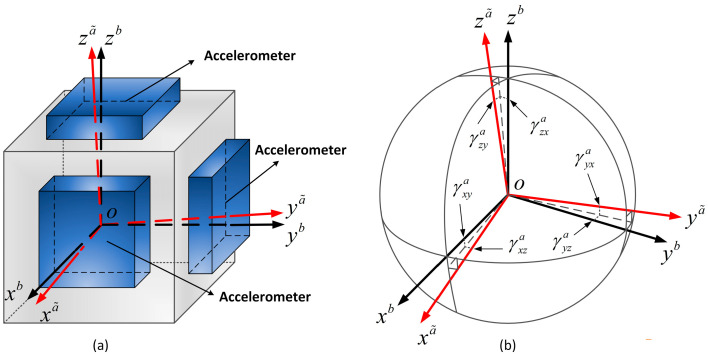
Installation error of the accelerometer assembly. (**a**) Installation diagrammatic drawing of the accelerometer assembly; (**b**) Installation errors between the accelerometer exes and b-frame exes.

Installation error matrix is a 3 × 3 matrix describing the accelerometer frame (or the gyroscope frame) to the body frame. It is the mathematical representation of the installation error. The accelerometer frame (a-frame) and the gyroscope frame (g-frame) coincide with the body frame (b-frame) in ideal condition. However, there are deviations in the actual installation. In the actual installation, the $\tilde{a}$-frame or $\tilde{g}$-frame deviates from the b-frame [26]. Suppose the origins of the $\tilde{a}$-frame, $\tilde{g}$-frame, and b-frame are coincident. Take the acceleration installation error modeling for an example. The actual installation of accelerometers is shown in Figure 1.

Figure 1 shows the actual installation frame of the accelerometer assembly. The three sensitive axes of the $\tilde{a}$-frame are not orthogonal. The three axes of the b-frame are orthogonal and $\gamma_{ij}^{a}(i,j=x,y,z,j=x,y,z)$ denotes installation error. The projections of the accelerometer measurements on the three axes of the b-frame are shown in Table 2.

Table 2 shows the output of the accelerometer. $N_{ij}^{b}(i=x,y,z,j=x,y,z)$ is the projection from the i-axis of the $\tilde{a}$-frame to the j-axis of the b-frame. $\gamma_{ij}^{a}(i=x,y,z,j=x,y,z)$ is the installation error of the accelerometer assembly. The specific force of the accelerometer assembly in the $\tilde{a}$-frame can be transformed into a specific force in the b-frame.

(1) $$N^{b}=C_{\tilde{a}}^{b}N^{\tilde{a}}$$

where $C_{\tilde{a}}^{b}$ represents the transformation matrix from the $\tilde{a}$-frame to the b-frame.

(2) $$C_{\tilde{a}}^{b}=\left[\begin{array}{ccc} \cos\gamma_{xz}^{a}\cos\gamma_{xy}^{a} & -\cos\gamma_{yx}^{a}\sin\gamma_{yz}^{a} & \sin\gamma_{zy}^{a}\\ \sin\gamma_{xz}^{a} & \cos\gamma_{yx}^{a}\cos\gamma_{yz}^{a} & -\cos\gamma_{zy}^{a}\sin\gamma_{zx}^{a}\\ -\cos\gamma_{xz}^{a}\sin\gamma_{xy}^{a} & \sin\gamma_{yx}^{a} & \cos\gamma_{zy}^{a}\cos\gamma_{zx}^{a} \end{array}\right]$$

where $\gamma_{ij}^{a}(i=x,y,z,j=x,y,z)$ is a small angle, $\cos\gamma_{ij}^{a}\approx 1$, and $\sin\gamma_{ij}^{a}\approx \gamma_{ij}^{a}$. The accelerometer transformation matrix $C_{\tilde{a}}^{b}$ can be written as:

(3) $$C_{\tilde{a}}^{b}=\left[\begin{array}{ccc} 1 & -\gamma_{yz}^{a} & \gamma_{zy}^{a}\\ \gamma_{xz}^{a} & 1 & -\gamma_{zx}^{a}\\ -\gamma_{xy}^{a} & \gamma_{yx}^{a} & 1 \end{array}\right]$$

The transformation relationship between $\tilde{a}$-frame and b-frame is

(4) $$\left[\begin{array}{c} x^{b}\\ y^{b}\\ z^{b} \end{array}\right]=C_{\tilde{a}}^{b}\left[\begin{array}{c} x^{\tilde{a}}\\ y^{\tilde{a}}\\ z^{\tilde{a}} \end{array}\right]$$

There is no installation error in the ideal condition. So, the a-frame and the b-frame are coincident. The transformation relationship is written as:

(5) $$\left[\begin{array}{c} x^{b}\\ y^{b}\\ z^{b} \end{array}\right]=C_{a}^{b}\left[\begin{array}{c} x^{a}\\ y^{a}\\ z^{a} \end{array}\right]=I_{3\times 3}\left[\begin{array}{c} x^{a}\\ y^{a}\\ z^{a} \end{array}\right]$$

The transformation matrix $C_{a}^{b}$ is a unit matrix ($C_{a}^{b}=I_{3\times 3}$). Equation (4) can also be rewritten as:

(6) $$\left[\begin{array}{c} x^{b}\\ y^{b}\\ z^{b} \end{array}\right]=C_{\tilde{a}}^{b}\left[\begin{array}{c} x^{\tilde{a}}\\ y^{\tilde{a}}\\ z^{\tilde{a}} \end{array}\right]=(I_{3\times 3}+\delta C_{a}^{b})\left[\begin{array}{c} x^{\tilde{a}}\\ y^{\tilde{a}}\\ z^{\tilde{a}} \end{array}\right]$$

$\delta C_{a}^{b}$ is the installation error matrix. It can be written as:

(7) $$\delta C_{a}^{b}=\left[\begin{array}{ccc} 0 & -\gamma_{yz}^{a} & \gamma_{zy}^{a}\\ \gamma_{xz}^{a} & 0 & -\gamma_{zx}^{a}\\ -\gamma_{xy}^{a} & \gamma_{yx}^{a} & 0 \end{array}\right]$$

Combine (5) and (6) together. $C_{\tilde{a}}^{b}$ is written as:

(8) $$C_{\tilde{a}}^{b}=C_{a}^{b}+\delta C_{a}^{b}$$

where $C_{\tilde{a}}^{b}$ represents the transformation matrix from the $\tilde{a}$-frame to the b-frame. $C_{a}^{b}$ represents the transformation matrix from the a-frame to the b-frame. $\delta C_{a}^{b}$ represents the installation error matrix.

Similarly, the derivation process of the gyroscope installation error model is the same as the accelerometer installation error model. Therefore, they have the same expression.

(9) $$\delta C_{g}^{b}=\left[\begin{array}{ccc} 0 & -\gamma_{yz}^{g} & \gamma_{zy}^{g}\\ \gamma_{xz}^{g} & 0 & -\gamma_{zx}^{g}\\ -\gamma_{xy}^{g} & \gamma_{yx}^{g} & 0 \end{array}\right]$$

The transformation matrixes $C_{\tilde{a}}^{b}$ and $C_{\tilde{g}}^{b}$ are as follows:

(10) $$\left\{\begin{array}{l} C_{\tilde{a}}^{b}=C_{a}^{b}+\delta C_{a}^{b}=I_{3\times 3}+\delta C_{a}^{b}\\ C_{\tilde{g}}^{b}=C_{g}^{b}+\delta C_{g}^{b}=I_{3\times 3}+\delta C_{g}^{b} \end{array}\right.$$

## 3. Polar Decomposition of Installation Error Matrix

In Section 2, the installation error is modeled for the accelerometer and gyroscope. The transformation matrix $C_{\tilde{a}(\tilde{g})}^{b}$ ($C_{\tilde{a}(\tilde{g})}^{b}$ represents the transformation matrix from the $\tilde{a}$-frame or the $\tilde{g}$-frame to the b-frame.) and the installation error matrix $\delta C_{a(g)}^{b}$ ($\delta C_{a(g)}^{b}$ represents the installation error matrix of the accelerometer or gyroscope.) are derived. $C_{\tilde{a}(\tilde{g})}^{b}$ is a direct transformation. In order to express this transformation more clearly, the polar decomposition method is introduced to divide the transformation into two steps. In this way, the compensation for the installation error also needs two steps.

The installation error is minimal. The rank of $C_{\tilde{a}(\tilde{g})}^{b}$ is three. $C_{\tilde{a}(\tilde{g})}^{b}$ is nonsingular, so it can perform singular value decomposition. According to singular value decomposition theory [27,28], $C_{\tilde{a}(\tilde{g})}^{b}$ can be decomposed as:

(11) $$C_{\tilde{a}(\tilde{g})}^{b}=U\Sigma V^{T}$$

where U represents the left singular matrix and V represents the right singular matrix. U and V are orthogonal matrixes. In Figure 1, the basis of the b-frame is a standard orthogonal basis. U and V are unit orthogonal matrixes.

V is a unit orthogonal matrix. Therefore, $VV^{T}=V^{T}V=I$. Substitute $VV^{T}$ into (11), and introduce the polar decomposition method [25]. Then

(12) $$U\Sigma V^{T}=UV^{T}V\Sigma V^{T}=(UV^{T})(V\Sigma V^{Τ})$$

Define $C_{b^{\prime }}^{b}=UV^{T}$ and $C_{\tilde{a}(\tilde{g})}^{b^{\prime }}=V\Sigma V^{T}$. Equation (11) can be written as:

(13) $$C_{\tilde{a}(\tilde{g})}^{b}=C_{b^{\prime }}^{b}C_{\tilde{a}(\tilde{g})}^{b^{\prime }}$$

where the $b^{\prime }$-frame is a rectangular Cartesian coordinate system.

According to (13), $C_{\tilde{a}(\tilde{g})}^{b}$ can be decomposed into $C_{\tilde{a}(\tilde{g})}^{b^{\prime }}$ and $C_{b^{\prime }}^{b}$ by polar decomposition. $C_{\tilde{a}(\tilde{g})}^{b^{\prime }}$ represents the transformation matrix from $\tilde{a}$-frame (or $\tilde{g}$-frame) to a rectangular cartesian coordinate system. $C_{b^{\prime }}^{b}$ represents the transformation matrix from the $b^{\prime }$-frame to the b-frame.

The transposition of $C_{\tilde{a}(\tilde{g})}^{b^{\prime }}$ is

(14) $${\left(C_{\tilde{a}(\tilde{g})}^{b^{\prime }}\right)}^{T}={\left(V\Sigma V^{T}\right)}^{T}=V\Sigma^{T}V^{T}=V\Sigma V^{T}=C_{\tilde{a}(\tilde{g})}^{b^{\prime }}$$

From the derivation of (14), it can be seen that $C_{\tilde{a}(\tilde{g})}^{b^{\prime }}$ is a symmetric matrix. The symmetric matrix $C_{\tilde{a}(\tilde{g})}^{b^{\prime }}$ represents the nonorthogonal properties of IMU. Suppose that the three nonorthogonal angles are $\mu_{x}$, $\mu_{y}$, and $\mu_{z}$.

(15) $$\mu ={\left[\begin{array}{ccc} \mu_{x} & \mu_{y} & \mu_{z} \end{array}\right]}^{T}$$

The column vector of $C_{\tilde{a}(\tilde{g})}^{b^{\prime }}$ is a unit vector. $C_{\tilde{a}(\tilde{g})}^{b^{\prime }}$ can be written as:

(16) $$C_{\tilde{a}(\tilde{g})}^{b^{\prime }}=\left[\begin{array}{ccc} \sqrt{1-\mu_{z}^{2}-\mu_{y}^{2}} & \mu_{z} & \mu_{y}\\ \mu_{z} & \sqrt{1-\mu_{z}^{2}-\mu_{x}^{2}} & \mu_{x}\\ \mu_{y} & \mu_{x} & \sqrt{1-\mu_{y}^{2}-\mu_{x}^{2}} \end{array}\right]$$

where $\mu_{x}$, $\mu_{y}$, and $\mu_{z}$ are all small angles, and the high-order infinitesimal can be ignored. Thus, $C_{\tilde{a}(\tilde{g})}^{b^{\prime }}$ can be written as:

(17) $$C_{\tilde{a}(\tilde{g})}^{b^{\prime }}\approx \left[\begin{array}{ccc} 1 & \mu_{z} & \mu_{y}\\ \mu_{z} & 1 & \mu_{x}\\ \mu_{y} & \mu_{x} & 1 \end{array}\right]=I+S(\mu )$$

where S(μ) is a symmetric matrix composed of three nonorthogonal angles.

$C_{b^{\prime }}^{b}$ is the transformation matrix from the $b^{\prime }$-frame to the b-frame. The orthogonal small-angle transformation matrix is a skew-symmetric matrix. Therefore, $C_{b^{\prime }}^{b}$ is a skew-symmetric matrix [29,30]. The three misalignment angles between the $b^{\prime }$-frame and the b-frame are $\eta_{x}$, $\eta_{y}$, and $\eta_{z}$.

(18) $$\eta ={\left[\begin{array}{ccc} \eta_{x} & \eta_{y} & \eta_{z} \end{array}\right]}^{T}$$

The three misalignment angles are all small angles, and the high-order infinitesimal can be ignored. Thus, $C_{b^{\prime }}^{b}$ is written as:

(19) $$C_{b^{\prime }}^{b}=I+\frac{\sin a}{a}(\eta \times )+\frac{1-\cos a}{a^{2}}{\left(\eta \times \right)}^{2}\approx I+(\eta \times )$$

where $a={\left\|\eta \right\|}_{2}=\sqrt{\left(\eta ,\eta \right)}$.

Through the above derivation, according to polar decomposition, the installation error matrix is divided into the product of two matrices. The frame is transformed twice. Substitute the transformation matrixes into (13) and ignore the small quantities of the second order and above the second order.

(20) $$C_{\tilde{a}(\tilde{g})}^{b}=C_{b^{\prime }}^{b}C_{\tilde{a}(\tilde{g})}^{b^{\prime }}=\left(I+(\eta \times )\right)\left(I+S(\mu )\right)\approx I+S(\mu )+(\eta \times )$$

Thus, $C_{\tilde{a}(\tilde{g})}^{b}$ can be written as:

(21) $$C_{\tilde{a}(\tilde{g})}^{b}=\left[\begin{array}{ccc} 1 & \mu_{z}-\eta_{z} & \mu_{y}+\eta_{y}\\ \mu_{z}+\eta_{z} & 1 & \mu_{x}-\eta_{x}\\ \mu_{y}-\eta_{y} & \mu_{x}+\eta_{x} & 1 \end{array}\right]$$

In some references [31,32,33], the matrix is directly decomposed into the sum of the symmetric matrix and skewed the symmetric matrix. In [29], the matrix is directly decomposed into the product of the symmetric matrix and the skew-symmetric matrix. The above derivation process also proves that this modeling method is feasible. In summary, the installation error matrix is decomposed into the product of two matrices by polar decomposition. The modeling in the geometric space requires two steps. The first step is to orthogonalize the nonorthogonal frame. The second step is to compensate for the misalignment of angles.

**Figure 2:**
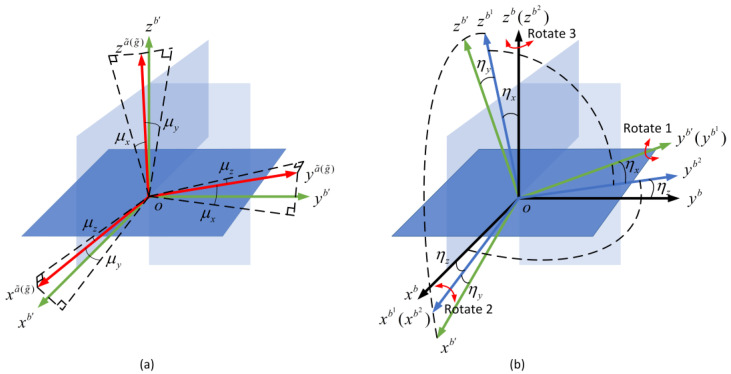
The transformation between two coordinate systems. (**a**) Orthogonalization of the nonorthogonal coordinate system; (**b**) Compensation of the misalignment angles.

In geometric space decomposing the installation error matrix by polar decomposition carries out two steps. The first step is the orthogonalization of the nonorthogonal coordinate system. It is shown in Figure 2a.

A nonorthogonal coordinate system is $o-x^{\tilde{a}(\tilde{g})}y^{\tilde{a}(\tilde{g})}z^{\tilde{a}(\tilde{g})}$, and $o-x^{b^{\prime }}y^{b^{\prime }}z^{b^{\prime }}$ is an orthogonal coordinate system. The essence of orthogonalization is to transform the nonorthogonal coordinate system into the orthogonal coordinate system. In Figure 2a, it transforms $o-x^{\tilde{a}(\tilde{g})}y^{\tilde{a}(\tilde{g})}z^{\tilde{a}(\tilde{g})}$ into $o-x^{b^{\prime }}y^{b^{\prime }}z^{b^{\prime }}$. The $b^{\prime }$-frame is an orthogonal coordinate system. There are misalignment angles between the $b^{\prime }$-frame and the b-frame. The misalignment relationship can be compensated by the transformation of the orthogonal small-angle transform [29,30]. The principle of orthogonal small-angle transform is shown in Figure 2b. In Figure 2b, the $b^{\prime }$-frame needs to rotate three times. The three rotations correspond to rotate one, rotate two, and rotate those. In this way, the orthogonalization and alignment of the IMU are completed. The IMU-frame and the b-frame are coincident.

$C_{\tilde{a}(\tilde{g})}^{b}$ is decomposed to a left singular matrix, right singular matrix, and singular value matrix by polar decomposition. After a series of matrix transformations, the final decomposition is the product of two matrices. This method of decomposing $C_{\tilde{a}(\tilde{g})}^{b}$ into the product of two matrices actually divides the installation error into two types. One type of installation error is a nonorthogonal error. It describes the three axes of the accelerometer (or gyroscope) assembly that are nonorthogonal. It cannot form a three-dimensional Cartesian coordinate system when the accelerometers are installed. Another type of installation error is a misalignment error. The b-frame is a fixed Cartesian coordinate system. Therefore, even $\tilde{a}$-frame or $\tilde{g}$-frame is an orthogonal installation, there may be misalignment errors relative to b-frame. This method decomposes the direct transformation into two steps in geometric space. The first step is the orthogonalization of the nonorthogonal coordinate system. The second step is to solve the problem of misalignment.

## 4. Calibration Experiment and Result Analysis

### 4.1. Calibration Experiment

**Figure 3:**
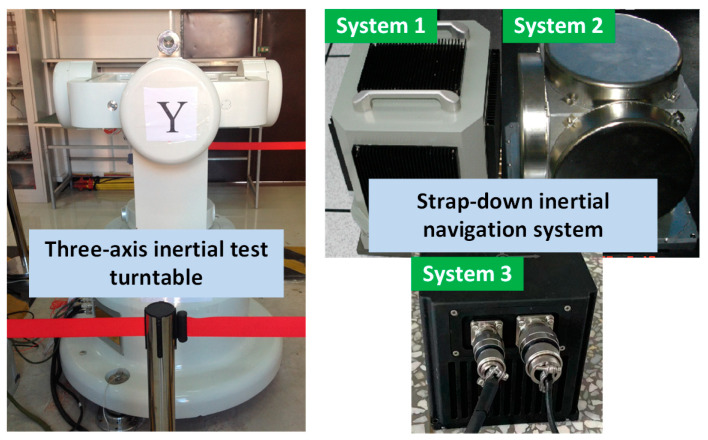
Three-axis inertial test turntable and 3 sets of SINS.

According to [7], three sets of SINS were calibrated by a three-axis inertial test turntable. Every system was calibrated twice based on the system-level calibration method. The SINS and the three-axis inertial test turntable are shown in Figure 3.

**Figure 4:**
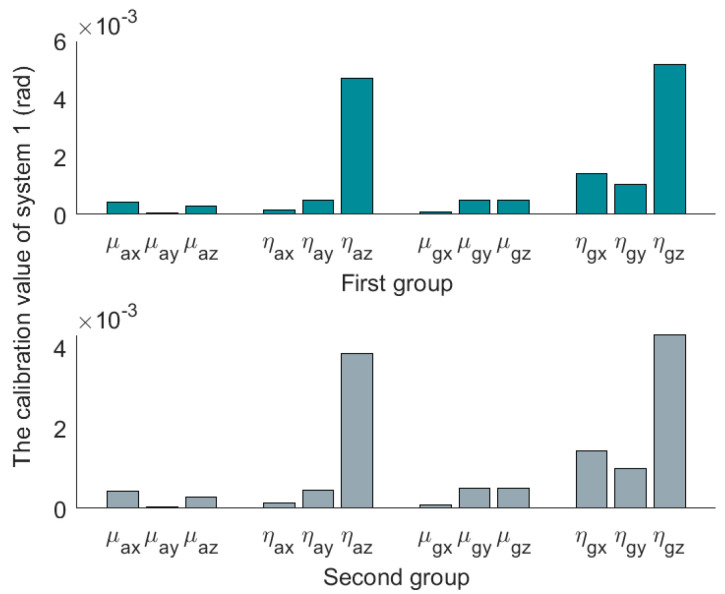
Absolute value of decomposition results of system 1.

**Figure 5:**
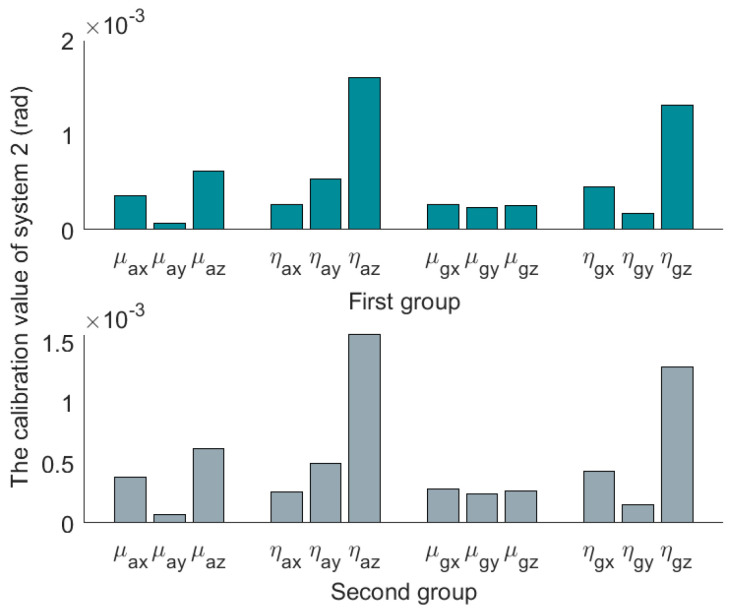
Absolute value of decomposition results of system 2.

**Figure 6:**
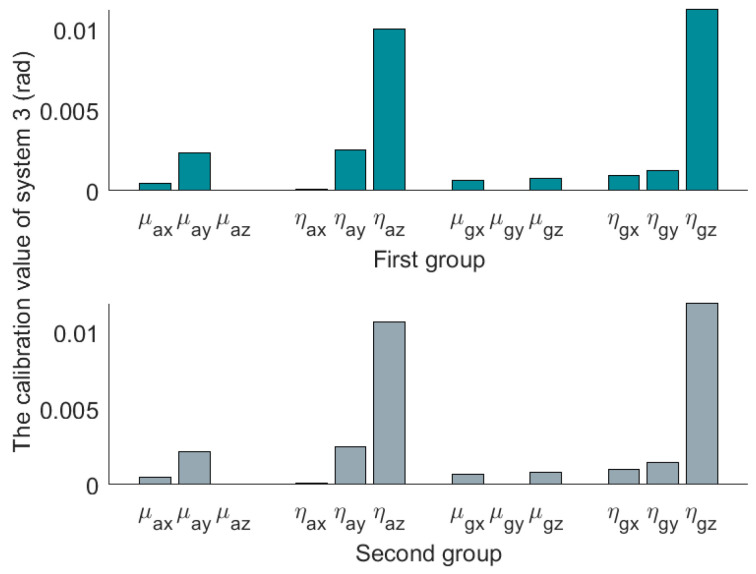
Absolute value of decomposition results of system 3.

The three sets of SINS calibration results are shown in Table 3. The first group is the result of the first calibration, and the second group is the result of the second calibration. The installation error matrixes are decomposed by polar decomposition in Table 3. After decomposition, we get three nonorthogonal angles and three misalignment angles. The nonorthogonal angles and the misalignment angles in the first calibration are represented by $\left[\begin{array}{ccc} \mu_{x1} & \mu_{y1} & \mu_{z1} \end{array}\right]$ and $\left[\begin{array}{ccc} \eta_{x1} & \eta_{y1} & \eta_{z1} \end{array}\right]$, and $\left[\begin{array}{ccc} \mu_{x2} & \mu_{y2} & \mu_{z2} \end{array}\right]$ and $\left[\begin{array}{ccc} \eta_{x2} & \eta_{y2} & \eta_{z2} \end{array}\right]$ represent the nonorthogonal angles and misalignment angles in the second calibration. The absolute values of the results are shown in Figure 4, Figure 5 and Figure 6.

### 4.2. Result Analysis

Compared to the calibration results in Figure 4, Figure 5 and Figure 6, it can be seen that the misalignment angle is larger than the nonorthogonal angle. To further analyze the two types of error angles, the $L_{\infty }$-norm and $L_{2}$-norm are introduced to compare them. Taking the nonorthogonal angle for example, its representations in the $L_{\infty }$-norm and $L_{2}$-norm are shown in (22).

(22) $$\left\{\begin{array}{l} {\left\|\mu \right\|}_{\infty }=\max\left\{\left|\mu_{x}\right|,\left|\mu_{y}\right|,\left|\mu_{z}\right|\right\}\\ {\left\|\mu \right\|}_{2}=\sqrt{{\left|\mu_{x}\right|}^{2}+{\left|\mu_{y}\right|}^{2}+{\left|\mu_{z}\right|}^{2}} \end{array}\right.$$

**Figure 7:**
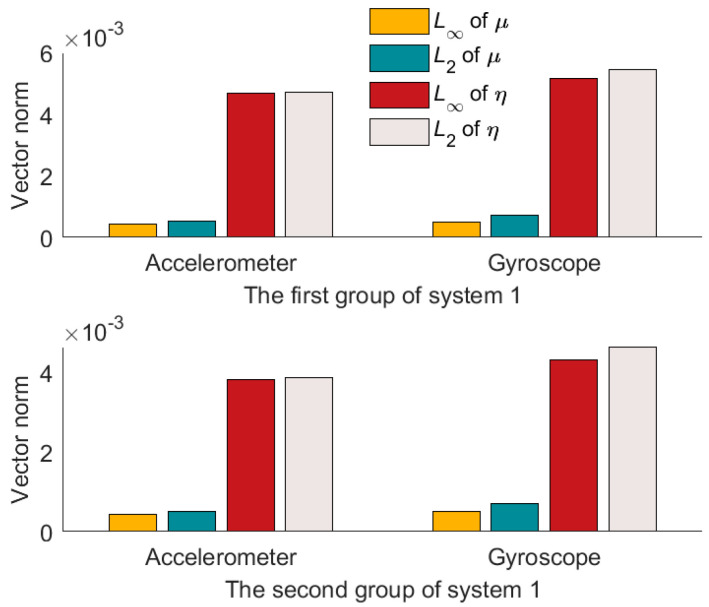
Results of two norm solutions for system 1.

**Figure 8:**
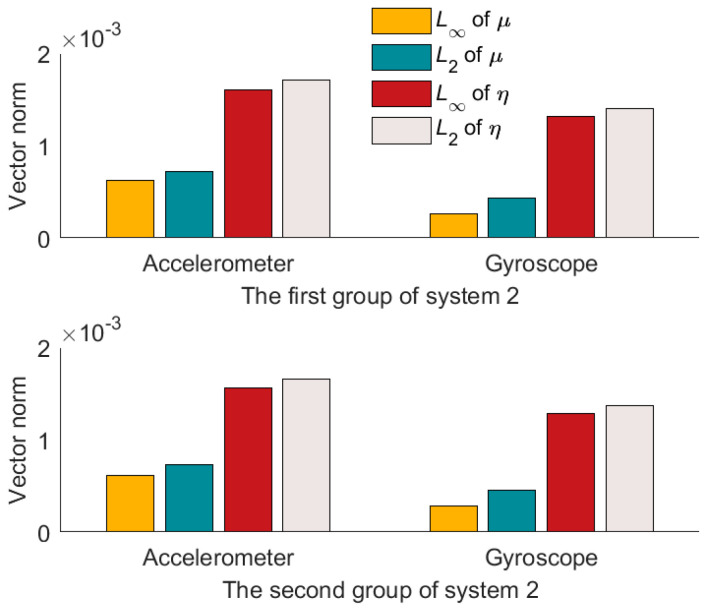
Results of two norm solutions for system 2.

**Figure 9:**
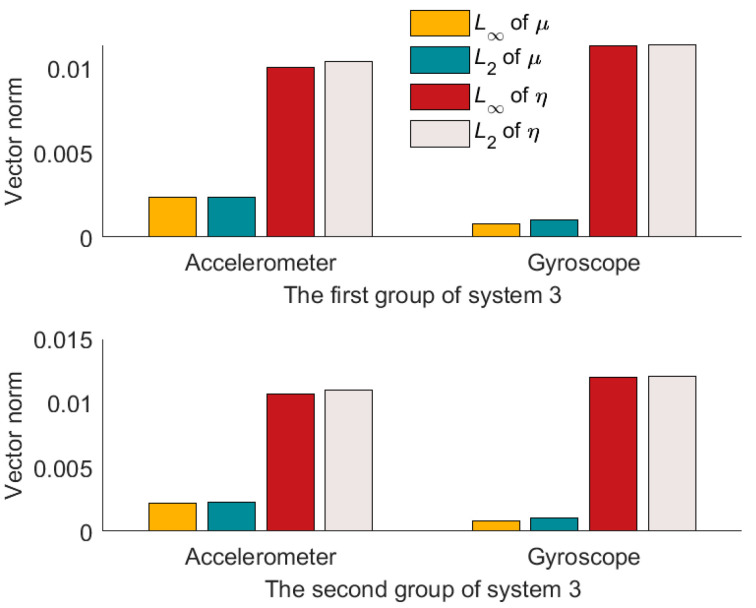
Results of two norm solutions for system 3.

According to (22), $L_{\infty }$-norm and $L_{2}$-norm are calculated for the two types of error angles. The results are shown in Table 4 and Table 5. And the $L_{\infty }$-norm values and $L_{2}$-norm values are drawn in Figure 7, Figure 8 and Figure 9.

From Figure 7, Figure 8 and Figure 9, we can see that the misalignment angles are larger than the nonorthogonal angles, ${\left\|\mu \right\|}_{\infty }<{\left\|\eta \right\|}_{\infty }$. ${\left\|\mu \right\|}_{2}<{\left\|\eta \right\|}_{2}$. Taking the result of $L_{2}$-norm as an example, the two calibration results of each system are averaged. In the three systems, the nonorthogonal errors of the accelerometer assembly are 12.13%, 42.88%, and 21.69% of the misalignment errors, respectively, and the nonorthogonal errors of the gyroscope assembly are 14.07%, 32.04%, and 8.67% of misalignment errors, respectively. Therefore, in the production process of IMU, more attention should be paid to the misalignment errors of IMU. That is the misalignment angles between the IMU coordinate system and the carrier coordinate system.

## 5. Static Navigation Experiment and Result Analysis

In order to save time and cost, the calibration model of the SINS is often simplified. In the traditional simplified models, the installation error matrix is often simplified as an upper triangular or lower triangular matrix. This will affect the navigation accuracy of SINS.

Through the analyses of installation error angles in Section 4, it is found that the misalignment error angles are greater than the nonorthogonal error angles in the three systems. Can we simplify the installation error matrix into an antisymmetric matrix composed of three misalignment angles? If so, is it better than the traditional simplified models? The paper will discuss and analyze the 48-h navigation experiments to verify these questions.

The simplified installation error model of the traditional lower triangular matrix [14,15,16,17,18] is:

(23) $$\delta C_{a(g)}^{b}=\left[\begin{array}{ccc} 0 & 0 & 0\\ \gamma_{xz}^{a(g)} & 0 & 0\\ -\gamma_{xy}^{a(g)} & \gamma_{yx}^{a(g)} & 0 \end{array}\right]$$

The simplified installation error model composed of the three misalignment angles is:

(24) $$\delta C_{a(g)}^{b}=\left[\begin{array}{ccc} 0 & -\eta_{a(g)z} & \eta_{a(g)y}\\ \eta_{a(g)z} & 0 & -\eta_{a(g)x}\\ -\eta_{a(g)y} & \eta_{a(g)x} & 0 \end{array}\right]$$

where $\delta C_{a(g)}^{b}$ is the installation error matrix of the accelerometer assembly (or gyroscope assembly). $\eta_{a(g)x}$, $\eta_{a(g)y}$, and $\eta_{a(g)z}$ are misalignment angles.

**Figure 10:**
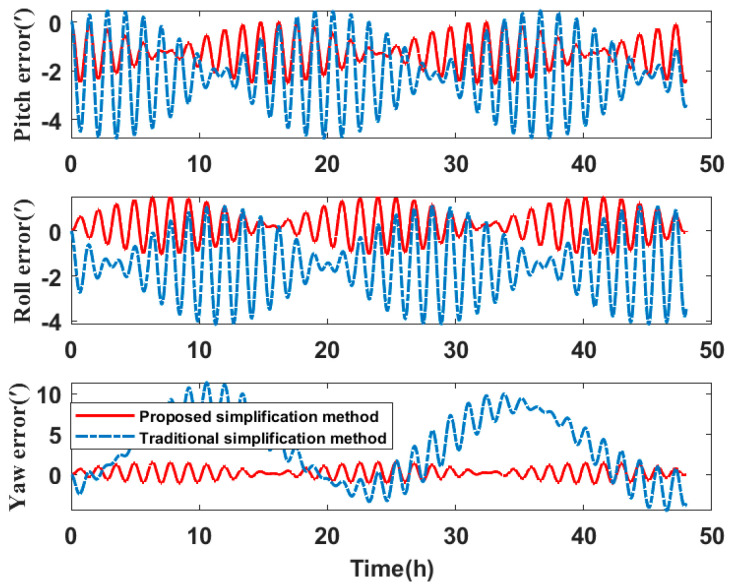
Attitude angle error of navigation simulation experiment results.

**Figure 11:**
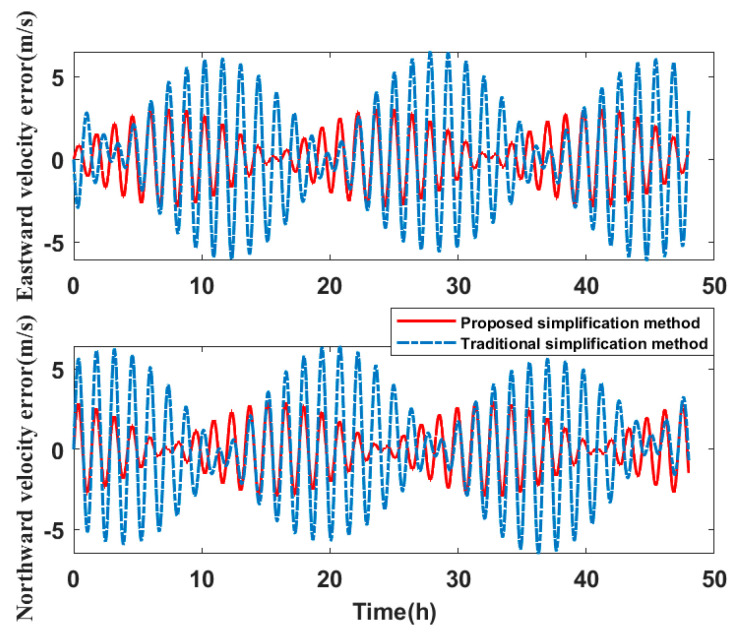
Velocity error of navigation simulation experiment results.

**Figure 12:**
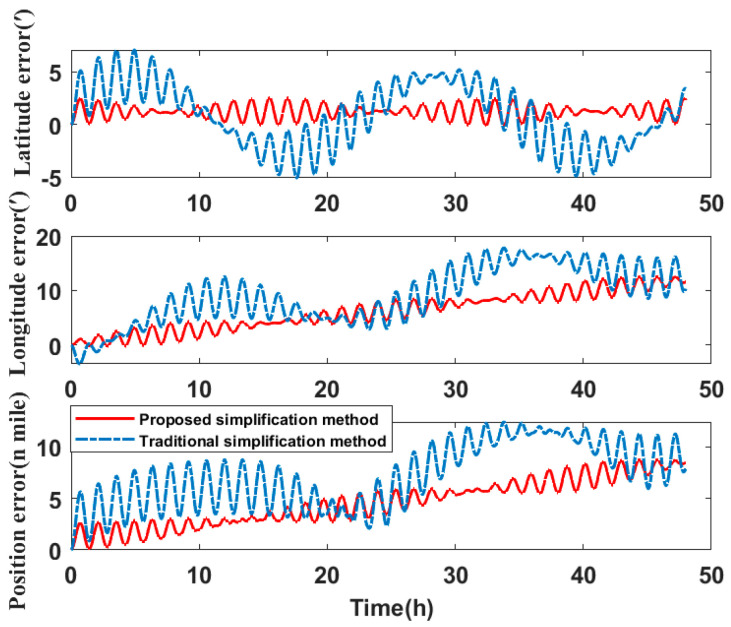
Position error of navigation simulation experiment results.

In comparing the simplified model of the three misalignment angles with the traditional simplified model of the lower triangular matrix, the experiments analyze the navigation errors caused by the installation errors. Ignore the influence of the other errors on SINS. Navigation simulation experiments are carried out for the three systems. The experimental results are shown in Table 6. We take system two as an example to show the navigation errors caused by the simplified models. The navigation results are shown in Figure 10, Figure 11 and Figure 12.

Compared to the navigation results of the systems in Table 6, the errors of the simplified model proposed in this paper are smaller than the error caused by the traditional simplified model in attitude, velocity, and position. The attitude accuracy of the systems is increased by 37.48–86.20%, the velocity accuracy is increased by 51.79–55.56%, and the position accuracy is increased by 21.94–76.37%.

Therefore, it can be concluded that if the misalignment angles of the SINS are larger than the nonorthogonal angles after singular value decomposition, the navigation errors caused by the simplified model proposed in this paper are less than the navigation errors caused by the traditional simplified model. The navigation errors of system three are the largest of the three systems. By analyzing the error calibration results in Table 3, the installation errors of system three are also much larger than that of system one and system two. The simplified calibration model causes large navigation errors. This directly shows that when the SINS installation error is large, the simplified model will also cause large navigation errors.

## 6. Conclusions

Aiming at the IMU calibration modeling of SINS, this paper proposes a new installation error model based on polar decomposition. This model divided the installation error into nonorthogonal angles and misalignment angles. The geometric transformation of the coordinate system was completed by the orthogonalization of the nonorthogonal coordinate system and the alignment of the misalignment coordinate system. Three sets of SINS were calibrated by a three-axis inertial test turntable. From the calibration results, we could find that the misalignment angles are all larger than the nonorthogonal angles. This indicates a direction for improving the IMU manufacturing level.

In order to reduce the influence of the simplified calibration model on navigation accuracy, a simplified model based on the misalignment angles is established. The calibration parameters of the three sets of SINS are substituted into 48 h navigation experiments for verification. The experiment results show that the navigation errors caused by the simplified model based on misalignment angles are smaller than those caused by the traditional simplified model of the lower triangular matrix. This provides a reference for the simplification of the SINS calibration model.

## Figures and Tables

**Table 1:** Frame definition.

Frames	Definition
b	The body frame
a	The accelerometer frame
g	The gyroscope frame
$\tilde{a}$	The accelerometer installation frame
$\tilde{g}$	The gyroscope installation frame

**Table 2:** Projections of accelerometer measurements.

*x*-Axis	*y*-Axis	*z*-Axis
$N_{xx}^{b}=N_{x}^{\tilde{a}}\cos\gamma_{xz}^{a}\cos\gamma_{xy}^{a}$	$N_{xy}^{b}=-N_{y}^{\tilde{a}}\cos\gamma_{yx}^{a}\sin\gamma_{yz}^{a}$	$N_{xz}^{b}=N_{z}^{\tilde{a}}\sin\gamma_{zy}^{a}$
$N_{yx}^{b}=N_{x}^{\tilde{a}}\sin\gamma_{xz}^{a}$	$N_{yy}^{b}=N_{y}^{\tilde{a}}\cos\gamma_{yx}^{a}\cos\gamma_{yz}^{a}$	$N_{yz}^{b}=-N_{z}^{\tilde{a}}\cos\gamma_{zy}^{a}\sin\gamma_{zx}^{a}$
$N_{zx}^{b}=-N_{x}^{\tilde{a}}\cos\gamma_{xz}^{a}\sin\gamma_{xy}^{a}$	$N_{zy}^{b}=N_{y}^{\tilde{a}}\sin\gamma_{yx}^{a}$	$N_{zz}^{b}=N_{z}^{\tilde{a}}\cos\gamma_{zy}^{a}\cos\gamma_{zx}^{a}$

**Table 3:** The calibration results of the installation error.

Group	System	Accelerometer Installation Error (Rad)	Gyroscope Installation Error (Rad)
First	1	${\left[\begin{array}{ccc} & 0.004957341 & 0.000536906\\ -0.004399740 & & -0.000577303\\ -0.000454405 & -0.000290616 & \end{array}\right]}_{3\times 3}$	${\left[\begin{array}{ccc} & 0.004664025 & 0.000545686\\ -0.005649291 & & 0.001499833\\ -0.001536893 & -0.001331234 & \end{array}\right]}_{3\times 3}$
	2	${\left[\begin{array}{ccc} & -0.002218500 & -0.000456892\\ 0.000982279 & & 0.000622934\\ 0.000600170 & 0.000089965 & \end{array}\right]}_{3\times 3}$	${\left[\begin{array}{ccc} & -0.001059488 & -0.000409414\\ 0.001572384 & & 0.000707237\\ -0.000063701 & -0.000187204 & \end{array}\right]}_{3\times 3}$
	3	${\left[\begin{array}{ccc} & -0.010023673 & -0.004856166\\ \;0.010128350 & & 0.000558046\\ 0.000163198 & 0.000323968 & \end{array}\right]}_{3\times 3}$	${\left[\begin{array}{ccc} & -0.010530742 & -0.001310682\\ 0.012094274 & & 0.000338906\\ 0.001226093 & -0.001616381 & \end{array}\right]}_{3\times 3}$
Second	1	${\left[\begin{array}{ccc} & 0.004109660 & 0.000489211\\ -0.003552206 & & -0.000573132\\ -0.000425666 & -0.000283261 & \end{array}\right]}_{3\times 3}$	${\left[\begin{array}{ccc} & 0.003820589 & 0.000494581\\ -0.004803553 & & 0.001503636\\ -0.001498021 & -0.001331522 & \end{array}\right]}_{3\times 3}$
	2	${\left[\begin{array}{ccc} & -0.002174314 & -0.000427485\\ 0.000948331 & & 0.000633462\\ 0.000563223 & 0.000128732 & \end{array}\right]}_{3\times 3}$	${\left[\begin{array}{ccc} & -0.001024323 & -0.000385621\\ 0.001545636 & & 0.000706864\\ -0.000091242 & -0.000149967 & \end{array}\right]}_{3\times 3}$
	3	${\left[\begin{array}{ccc} & -0.010654688 & -0.004695300\\ 0.010764496 & & 0.000577666\\ 0.000312103 & 0.000366105 & \end{array}\right]}_{3\times 3}$	${\left[\begin{array}{ccc} & -0.011171775 & -0.001462344\\ 0.012748961 & & 0.000360389\\ 0.001395436 & -0.001673174 & \end{array}\right]}_{3\times 3}$

**Table 4:** The $L_{\infty }$-norm of nonorthogonal and misalignment errors.

Systems	Sensors	First Group		Second Group	
		*μ* (×10^−3^)	*η* (×10^−3^)	*μ* (×10^−3^)	*η* (×10^−3^)
1	Acc	0.433959	4.678540	0.428196	3.830930
	Gyro	0.495604	5.156660	0.501720	4.312070
2	Acc	0.618111	1.600390	0.612992	1.561320
	Gyro	0.260017	1.315940	0.278448	1.284980
3	Acc	2.346480	10.076000	2.191600	10.709600
	Gyro	0.781766	11.312500	0.788593	11.960400

**Table 5:** The $L_{2}$-norm of nonorthogonal and misalignment errors.

Systems	Sensors	First Group		Second Group	
		*μ* (×10^−3^)	*η* (×10^−3^)	*μ* (×10^−3^)	*η* (×10^−3^)
1	Acc	0.517447	4.706905	0.511908	3.860865
	Gyro	0.703859	5.447858	0.707590	4.647160
2	Acc	0.717112	1.706343	0.724983	1.657342
	Gyro	0.435125	1.400565	0.449806	1.362489
3	Acc	2.388136	10.384506	2.242499	10.998874
	Gyro	1.010412	11.425290	1.026571	12.088289

**Table 6:** Experimental results.

Parameters	Navigation Errors of Sys. 1		Navigation Errors of Sys. 2		Navigation Errors of Sys. 3	
	Proposed Method	Traditional Method	Proposed Method	Traditional Method	Proposed Method	Traditional Method
Pitch (′)	2.977	4.762	2.550	4.787	9.519	18.623
Roll (′)	1.621	4.638	1.526	4.159	15.755	32.9173
Yaw (′)	8.341	33.899	1.569	11.367	20.614	81.979
Eastward velocity (m/s)	3.628	7.526	3.042	6.502	18.579	40.372
Northward velocity (m/s)	3.722	7.934	2.956	6.505	18.494	41.618
Longitude (′)	5.356	18.779	2.538	7.096	11.296	46.546
Latitude (′)	9.182	38.861	12.520	17.814	50.158	64.255
Position (n mile)	6.888	27.154	8.751	12.429	34.968	46.768

## Data Availability

The data that support the findings of this study are available from the corresponding author upon reasonable request.
